# A Global Bibliometric Analysis of the Top 100 Most Cited Articles on Carotid Body Tumors

**DOI:** 10.7759/cureus.54754

**Published:** 2024-02-23

**Authors:** Abdullah Abdullah, Abdulaziz Hamzah, Ali S Alsudais, Raghad S Alzahrani, Hatem Souror, Gutaybah S Alqarni, Afnan A Ashqar, Yousef H Hemeq, Omar Dakkak

**Affiliations:** 1 Department of Surgery, Faculty of Medicine, King Abdulaziz University, Jeddah, SAU; 2 College of Medicine, King Saud bin Abdulaziz University for Health Sciences, Jeddah, SAU; 3 Department of Research, King Abdullah International Medical Research Center, Jeddah, SAU; 4 College of Medicine, University of Jeddah, Jeddah, SAU; 5 College of Medicine, Batterjee Medical College, Jeddah, SAU; 6 Department of Surgery (Vascular Surgery), International Medical Center Hospital, Jeddah, SAU

**Keywords:** vascular surgery, chemodectoma, carotid paraganglioma, carotid body tumor, bibliometric analysis

## Abstract

The carotid body, situated at the common carotid artery bifurcation, comprises specialized glomus cells responsible for sensing blood oxygen, carbon dioxide, pH, and temperature changes, crucial for regulating breathing and maintaining oxygen homeostasis. Carotid body tumors (CBTs), arising from these cells, are rare, representing only 0.5% of head and neck tumors, often presenting as benign, slow-growing, vascularized masses. In February 2023, this bibliometric analysis was conducted, which involved screening 1733 articles from the Web of Science database. The screening process was based on citation count, and articles were selected for inclusion based on specific criteria that focused on CBTs located within the carotid bifurcation. Rigorous selection involved independent screening and data extraction by four authors. The top 100 articles, published between 1948 and 2019, totaled 6623 citations and were authored by 98 unique first authors from 22 countries and 77 institutions, spanning 42 journals. Treatment articles were the predominant category, comprising 49% of the literature. This analysis offers insights into publication trends, identifies literature gaps, and outlines areas of research focus, providing a valuable resource to guide future studies on CBTs.

## Introduction and background

The carotid body is a chemoreceptor located at the bifurcation of the common carotid artery in the neck. It consists of clusters of specialized cells called glomus cells, which sense changes in the oxygen and carbon dioxide levels in the blood. The carotid body also responds to changes in pH and temperature and plays a crucial role in regulating breathing and maintaining oxygen homeostasis. Moreover, it is innervated by the glossopharyngeal nerve (cranial nerve IX) and sends signals to the respiratory centers in the brainstem, which in turn modulates breathing and cardiovascular function [1].

Carotid body tumors (CBTs) are rare neoplasms that arise from the glomus cells of the carotid body. Representing only 0.5% of the total head and neck tumors, CBTs are the most common type of head and neck paragangliomas, followed by jugular foramen and vagal tumors. CBTs are usually benign with slow-growing, highly vascularized masses. Although the majority of CBTs present with a painless neck mass, they can cause pain, hoarseness, difficulty swallowing, and cranial nerve palsies if they grow large enough to compress adjacent structures. However, in some cases, CBTs can be malignant and metastasize to other parts of the body [2,3].

The earliest descriptions of CBTs can be traced back to the observations of Swiss anatomist von Haller during the 18th century. It was not until 1862 that Luschka provided the first microscopic image of CBT structure. Surgical attempts to remove CBTs have been documented since 1880 when Riegner made the first unsuccessful attempt. Subsequently, in 1886, Maydl successfully resected the carotid bifurcation, but the patient suffered from postoperative complications of hemiplegia, facial paralysis, and aphasia. Finally, in 1889, Albert achieved the first successful excision of a CBT without ligating the carotid vessels [4].

Bibliometric analysis has gained increasing recognition and acceptance as a research method within the scientific community. It enables the evaluation of the influence of published articles in a particular field and facilitates the identification of patterns and trends in publication evolution. Furthermore, it serves as a valuable tool for identifying gaps in scientific knowledge within a given discipline, guiding future research efforts [5]. Previous bibliometric analyses have been published in the field of vascular surgery to identify the impact, research trends, and developments in topics such as endovascular treatments [6,7], peripheral vascular disease [8], ankle-arm index [9], machine learning [10], and education [11]. In other fields, such as neurosurgery, bibliometric analysis is conducted on various topics, such as central nervous system hemangioblastomas and neurofibromatosis [12,13]. 

To the best of our knowledge, no prior publication has undertaken a bibliometric analysis specifically targeting CBTs. Consequently, this study seeks to offer a comprehensive review of the most impactful publications within this domain by analyzing the 100 most cited articles on CBTs. Through this analysis, we aim to delineate publication trends, identify research hotspots, and pinpoint existing gaps in the literature. Our objective is to furnish valuable insights into the top articles in CBT research, with the intention of informing and guiding future global multicenter research initiatives in this field.

## Review

Methodology

The study aimed to conduct a bibliometric analysis of the top 100 most cited articles on CBTs. In February 2023, the Web of Science (WoS) was screened for articles discussing CBTs. Title and keyword search was conducted using the following terms: "carotid body tumor," "carotid tumor," "carotid paraganglioma," and "chemodectoma." The search outcome was a total of 1733 articles. The articles were arranged based on citation count (CC) from highest to lowest and were extracted. Articles mainly discussing CBTs were included. Articles predominantly discussing carotid body-like tumors, CBTs outside of carotid bifurcation, and topics other than CBTsCC: citation count; CY: citation per year and veterinary articles were excluded. Also, articles whose full text was inaccessible online were excluded.

Selection Process and Statistical Analysis 

The study employed a rigorous selection process where two independent authors (Raghad S. Alzahrani and Ali S. Alsudais) conducted a comprehensive review of the search results' titles, abstracts, and full texts to identify the included papers. The process involved thoroughly screening 128 articles, with the top 100 being selected. Subsequently, two additional independent authors (Afnan A. Ashqar and Yousef H. Hemeq) scrutinized the included articles and extracted all pertinent data of interest. Any discrepancies that emerged during the process were resolved through a mutual agreement among all the authors.

Data on various parameters, including the title, year of publication, journal, CC, first author name, country, and institute, were gathered and subsequently classified into discrete categories of natural history/disease characteristics, treatment, radiology, histopathology/genetics, and review articles. The natural history/disease characteristics category encompassed articles that provided insights into the natural history, prognosis, clinical presentation, disease progression, and forms of CBTs. The treatment category comprised articles that dealt with different management aspects, such as surgery and pre-op embolization and their associated complications. The radiology category consisted of articles that discussed radiological classifications, various imaging modalities, and the role of radiological assessment. The histopathology/genetics category included articles that examined the microscopic characteristics of CBTs and their genetic predisposition. Finally, the review category includes articles that cover two or more aspects of CBTs without predominantly discussing a specific focus.

Data collection and statistical analysis were conducted using Microsoft Excel, Google Sheets, and JMP®, Version 15 (SAS Institute Inc., Cary, NC, 1989-2023).

Results

Search Results 

Our initial search resulted in 1733 articles with a total CC of 23,775 and an average citation of 13.72 times per paper. The top 100 most cited articles that met our criteria had a total number of citations of 6623, averaging 66.23 (50.66 standard deviation) per article and ranging from 37 to 462 citations. Ninety-eight unique first authors published them in 42 different journals from 1948 to 2019, 22 countries, and 77 institutions (Table 1).

**Table 1:** Complete list of the top 100 most cited articles in carotid body tumors #: serial number; YoP: year of publication; CC: citation count; CY: citation per year; USA: United States of America; UK: United Kingdom

#	YoP	Authors	Title	Journal	Category	Country	CC	CY
1	1971	Shamblin et al.	Carotid body tumor (chemodectoma). Clinicopathologic analysis of ninety cases [4]	The American Journal of Surgery	Histopathology/genetics	USA	462	8.88
2	1950	Lattes	Nonchromaffin paraganglioma of ganglion nodosum, carotid body, and aortic-arch bodies [14]	Cancer	Natural history/disease characteristics	USA	212	2.90
3	1980	Grufferman et al.	Familial carotid body tumors: case report and epidemiologic review [15]	Cancer	Natural history/disease characteristics	USA	192	4.47
4	2000	Jansen et al.	Estimation of growth rate in patients with head and neck paragangliomas influences the treatment proposal [16]	Cancer	Natural history/disease characteristics	Netherlands	175	7.61
5	2007	Sajid et al.	A multicenter review of carotid body tumour management [17]	European Journal of Vascular and Endovascular Surgery	Treatment	UK	153	9.56
6	1995	Netterville et al.	Carotid body tumors: a review of 30 patients with 46 tumors [18]	The Laryngoscope	Treatment	USA	123	4.39
7	1998	Rodríguez-Cuevas et al.	Carotid body tumors in inhabitants of altitudes higher than 2000 meters above sea level [19]	Head & Neck: Journal for the Sciences and Specialties of the Head and Neck	Natural history/disease characteristics	Mexico	116	4.64
8	2005	Luna-Ortiz et al.	Carotid body tumors: review of a 20-year experience [20]	Oral Oncology	Natural history/disease characteristics	Mexico	110	6.11
9	1988	Hallett et al.	Trends in neurovascular complications of surgical management for carotid body and cervical paragangliomas: a fifty-year experience with 153 tumors [21]	Journal of Vascular Surgery	Treatment	USA	101	2.89
10	1948	LeCompte	Tumors of the carotid body [22]	The American Journal of Pathology	Histopathology/genetics	USA	97	1.29
11	1996	Litle et al.	Preoperative embolization of carotid body tumors: when is it appropriate? [23]	Annals of Vascular Surgery	Treatment	USA	93	3.44
12	2001	Hinerman et al.	Definitive radiotherapy in the management of chemodectomas arising in the temporal bone, carotid body, and glomus vagale [24]	Head & Neck: Journal for the Sciences and Specialties of the Head and Neck	Treatment	USA	91	4.14
13	1982	Parry et al.	Carotid body tumors in humans: genetics and epidemiology [25]	Journal of the National Cancer Institute	Histopathology/genetics	USA	89	2.17
14	1986	Dickinson et al.	Carotid body tumour: 30 years experience [26]	British Journal of Surgery	Treatment	UK	88	2.38
15	1950	Monro	The natural history of carotid body tumours and their diagnosis and treatment; with a report of five cases [27]	British Journal of Surgery	Natural history/disease characteristics	UK	86	1.18
16	2012	Power et al.	Impact of preoperative embolization on outcomes of carotid body tumor resections [28]	Journal of Vascular Surgery	Treatment	USA	84	7.64
17	1998	Westerband et al.	Current trends in the detection and management of carotid body tumors [29]	Journal of Vascular Surgery	Review	USA	83	3.32
18	2016	Moore et al.	Head and neck paragangliomas: an update on evaluation and management [30]	Otolaryngology-Head and Neck Surgery	Review	USA	82	11.71
19	1954	Romanski	Chemodectoma (non-chromaffinic paraganglioma) of the carotid body with distant metastases; with illustrative case [31]	The American Journal of Pathology	Histopathology/genetics	USA	81	1.17
20	2000	Wang et al.	Surgical management of carotid body tumors [32]	Otolaryngology-Head and Neck Surgery	Treatment	USA	81	3.52
21	1992	Powell et al.	Chemodectoma of the head and neck: results of treatment in 84 patients [33]	International Journal of Radiation Oncology*Biology*Physics	Treatment	UK	76	2.45
22	1966	Staats et al.	Carotid body tumors, benign and malignant [34]	The Laryngoscope	Review	USA	71	1.25
23	1981	Lees et al.	Tumors of the carotid body. Experience with 41 operative cases [35]	The American Journal of Surgery	Treatment	USA	68	1.62
24	2006	Knight et al.	Current concepts for the surgical management of carotid body tumor [36]	The American Journal of Surgery	Treatment	USA	67	3.94
25	2008	Arya et al.	Carotid body tumors: objective criteria to predict the Shamblin group on MR imaging [37]	American Journal of Neuroradiology	Radiology	India	67	4.47
26	1973	Martin et al.	Carotid body tumors: a 16-year follow-up of seven malignant cases [38]	Southern Medical Journal	Natural history/disease characteristics	USA	65	1.30
27	2008	Makeieff et al.	Surgical management of carotid body tumors [39]	Annals of Surgical Oncology	Treatment	France	65	4.33
28	2010	Kruger et al.	Important observations made managing carotid body tumors during a 25-year experience [40]	Journal of Vascular Surgery	Treatment	Australia	65	5.00
29	2017	Williams	Paragangliomas of the head and neck: an overview from diagnosis to genetics [41]	Head and Neck Pathology	Review	USA	65	10.83
30	1953	Pettet et al.	Carotid body tumors (chemodectomas) [42]	Annals of Surgery	Review	USA	63	0.90
31	1997	Muhm et al.	Diagnostic and therapeutic approaches to carotid body tumors. Review of 24 patients [43]	Archives of Surgery	Review	Austria	63	2.42
32	2003	Timmers et al.	Baroreflex and chemoreflex function after bilateral carotid body tumor resection [44]	Journal of Hypertension	Treatment	Netherlands	61	3.05
33	2006	Luna-Ortiz et al.	Does Shamblin's classification predict postoperative morbidity in carotid body tumors? A proposal to modify Shamblin's classification [45]	European Archives of Oto-Rhino-Laryngology	Treatment	Mexico	61	3.59
34	1988	Nora et al.	Surgical resection of carotid body tumors: long-term survival, recurrence, and metastasis [46]	Mayo Clinic Proceedings	Treatment	Canada	60	1.71
35	1971	Capella and Solcia	Optical and electron microscopical study of cytoplasmic granules in human carotid body, carotid body tumours and glomus jugulare tumours [47]	Virchows Archiv	Histopathology/genetics	Italy	59	1.13
36	2001	Badenhop et al.	Novel mutations in the SDHD gene in pedigrees with familial carotid body paraganglioma and sensorineural hearing loss [48]	Genes, Chromosomes and Cancer	Histopathology/genetics	Australia	58	2.64
37	2010	Lim et al.	Surgical treatment of carotid body paragangliomas: outcomes and complications according to the Shamblin classification [49]	Clinical and Experimental Otorhinolaryngology	Treatment	Korea	58	4.46
38	1980	Schick et al.	Arterial catheter embolization followed by surgery for large chemodectoma [50]	Surgery	Treatment	USA	57	1.33
39	1963	Rush	Familial bilateral carotid body tumors [51]	Annals of Surgery	Histopathology/genetics	USA	57	0.95
40	1977	Gaylis and Mieny	The incidence of malignancy in carotid body tumours [52]	British Journal of Surgery	Review	South Africa	56	1.22
41	2002	Elshaikh et al.	Recurrent head-and-neck chemodectomas: a comparison of surgical and radiotherapeutic results [53]	International Journal of Radiation Oncology*Biology*Physics	Treatment	USA	56	2.67
42	1953	Morfit et al.	Carotid body tumors; report of twelve cases, including one case with proved visceral dissemination [54]	AMA Archives of Surgery	Natural history/disease characteristics	USA	55	0.79
43	1967	Farr	Carotid body tumors. A thirty year experience at Memorial Hospital [55]	The American Journal of Surgery	Treatment	USA	55	0.98
44	1986	Meyer et al.	Carotid body tumors: a subject review and suggested surgical approach [56]	Journal of Neurosurgery	Treatment	USA	55	1.49
45	2001	van der Mey et al.	Management of carotid body tumors [57]	Otolaryngologic Clinics of North America	Treatment	Netherlands	55	2.50
46	1959	Sessions et al.	Surgical experiences with tumors of the carotid body, glomus jugulare and retroperitoneal nonchromaffin paraganglia [58]	Annals of Surgery	Treatment	USA	54	0.84
47	1967	Toker	Ultrastructure of a chemodectoma [59]	Cancer	Histopathology/genetics	USA	54	0.96
48	1983	Padberg et al.	Carotid body tumor: the Lahey Clinic experience [60]	The American Journal of Surgery	Review	USA	54	1.35
49	1985	Zbaren and Lehmann	Carotid body paraganglioma with metastases [61]	The Laryngoscope	Natural history/disease characteristics	Switzerland	54	1.42
50	1988	Kimura et al.	Immunohistochemical study of chromogranin in 100 cases of pheochromocytoma, carotid body tumour, medullary thyroid carcinoma and carcinoid tumour [62]	Virchows Archiv	Histopathology/genetics	Japan	53	1.51
51	2017	Kim et al.	New predictors of complications in carotid body tumor resection [63]	Journal of Vascular Surgery	Treatment	USA	53	8.83
52	1968	Chambers and Mahoney	Carotid body tumors [64]	The American Journal of Surgery	Treatment	USA	52	0.95
53	1980	Farr	Carotid body tumors: a 40-year study [65]	CA: A Cancer Journal for Clinicians	Review	USA	52	1.21
54	1992	Williams et al.	Carotid body tumor [66]	Archives of Surgery	Treatment	USA	52	1.68
55	2002	Baysal and Myers	Etiopathogenesis and clinical presentation of carotid body tumors [67]	Microscopy Research and Technique	Natural history/disease characteristics	USA	52	2.48
56	2000	De Toma et al.	Baroreflex failure syndrome after bilateral excision of carotid body tumors: an underestimated problem [68]	Journal of Vascular Surgery	Treatment	Italy	51	2.22
57	2008	van der Bogt et al.	Resection of carotid body tumors: results of an evolving surgical technique [69]	Annals of Surgery	Treatment	Netherlands	50	3.33
58	2014	Suárez et al.	Carotid body paragangliomas: a systematic study on management with surgery and radiotherapy [70]	European Archives of Oto-Rhino-Laryngology	Treatment	Spain	50	5.56
59	1967	Hamberger et al.	Malignant catecholamine-producing tumour of the carotid body [71]	Acta Pathologica Microbiologica Scandinavica	Histopathology/genetics	Sweden	49	0.88
60	1989	McPherson et al.	Carotid body tumours and other cervical paragangliomas: diagnosis and management in 25 patients [72]	British Journal of Surgery	Treatment	UK	49	1.44
61	2001	Drovdlic et al.	Proportion of heritable paraganglioma cases and associated clinical characteristics [73]	The Laryngoscope	Histopathology/genetics	USA	49	2.23
62	1958	Byrne	Carotid body and allied tumors [74]	The American Journal of Surgery	Review	USA	48	0.74
63	1963	Fanning et al.	Metastatic carotid body tumor: report of a case with review of the literature [75]	JAMA-Journal of the American Medical Association	Natural history/disease characteristics	USA	48	0.80
64	1974	Chedid and Jao	Hereditary tumors of the carotid bodies and chronic obstructive pulmonary disease [76]	Cancer	Natural history/disease characteristics	USA	48	0.98
65	1990	Valdagni and Amichetti	Radiation therapy of carotid body tumors [77]	American Journal of Clinical Oncology	Treatment	Italy	48	1.45
66	1990	Barnes and Taylor	Carotid body paragangliomas. A clinicopathologic and DNA analysis of 13 tumors [78]	Archives of Otolaryngology	Histopathology/genetics	USA	48	1.45
67	1995	Anand et al.	Management of the internal carotid artery during carotid body tumor surgery [79]	The Laryngoscope	Treatment	USA	48	1.71
68	2001	Plukker et al.	Outcome of surgical treatment for carotid body paraganglioma [80]	British Journal of Surgery	Treatment	Netherlands	48	2.18
69	1992	Derchi et al.	Carotid body tumors: US evaluation [81]	Radiology	Radiology	Italy	47	1.52
70	2016	Abu-Ghanem et al.	Impact of preoperative embolization on the outcomes of carotid body tumor surgery: a meta-analysis and review of the literature [82]	Head & Neck: Journal for the Sciences and Specialties of the Head and Neck	Treatment	Israel	47	6.71
71	1974	Sato et al.	Concurrence of carotid body tumor and pheochromocytoma [83]	Cancer	Natural history/disease characteristics	Japan	46	0.94
72	2001	Kafie and Freischlag	Carotid body tumors: the role of preoperative embolization [84]	Annals of Surgery	Treatment	USA	46	2.09
73	1961	Costero and Barroso-Moguel	Structure of the carotid body tumor [85]	The American Journal of Pathology	Histopathology/genetics	Mexico	45	0.73
74	1965	Conley	The carotid body tumor. A review of 29 cases [86]	Archives of Otolaryngology	Review	USA	45	0.78
75	1968	Albores-Saavedra and Durán	Association of thyroid carcinoma and chemodectoma [87]	The American Journal of Surgery	Natural history/disease characteristics	Mexico	45	0.82
76	1948	MacComb	Carotid body tumors [88]	Annals of Surgery	Review	USA	45	0.6
77	1988	Pacheco-Ojeda et al.	Carotid body tumors at high altitudes: Quito, Ecuador, 1987 [89]	World Journal of Surgery	Review	Ecuador	44	1.26
78	2019	Robertson et al.	A systematic review and meta-analysis of the presentation and surgical management of patients with carotid body tumours [90]	European Journal of Vascular and Endovascular Surgery	Treatment	UK	44	11.00
79	2010	Zeitler et al.	Preoperative embolization in carotid body tumor surgery: is it required? [91]	Annals of Otology, Rhinology & Laryngology	Treatment	USA	43	3.31
80	1990	Miyazaki et al.	Resection of high-cervical paraganglioma with cervical-to-petrous internal carotid artery saphenous vein bypass. Report of two cases [92]	Journal of Neurosurgery	Treatment	Japan	42	1.27
81	1992	Massey and Wallner	Treatment of metastatic chemodectoma [93]	Cancer	Treatment	USA	42	1.35
82	1988	Ward et al.	Embolization: an adjunctive measure for removal of carotid body tumors [94]	The Laryngoscope	Treatment	USA	41	1.17
83	2001	Thabet and Kotob	Cervical paragangliomas: diagnosis, management and complications [95]	The Journal of Laryngology & Otology	Treatment	Egypt	41	1.86
84	2013	Fruhmann et al.	Paraganglioma of the carotid body: treatment strategy and SDH-gene mutations [96]	European Journal of Vascular and Endovascular Surgery	Treatment	Austria	41	4.10
85	2015	Jackson et al.	The effects of preoperative embolization on carotid body paraganglioma surgery: a systematic review and meta-analysis [97]	Otolaryngology-Head and Neck Surgery	Treatment	USA	41	5.13
86	2016	Davila et al.	Current surgical management of carotid body tumors [98]	Journal of Vascular Surgery	Treatment	USA	41	5.86
87	1970	Wilson	Carotid body tumors. Familial and bilateral [99]	Annals of Surgery	Natural history/disease characteristics	USA	40	0.75
88	1983	Borges et al.	Carotid body tumors managed with preoperative embolization [100]	Journal of Neurosurgery	Treatment	USA	40	1.00
89	2000	Mafee et al.	Glomus faciale, glomus jugulare, glomus tympanicum, glomus vagale, carotid body tumors, and simulating lesions. Role of MR imaging [101]	Radiologic Clinics of North America	Radiology	USA	40	1.74
90	1963	Reese et al.	Malignant carotid body tumors: report of a case [102]	Annals of Surgery	Natural history/disease characteristics	USA	39	0.65
91	1948	Donald and Crile	Tumors of the carotid body [103]	The American Journal of Surgery	Treatment	USA	38	0.51
92	1950	Lewison and Weinberg	Carotid body tumors: a case report of bilateral carotid body tumors with an unusual family incidence [104]	Surgery	Natural history/disease characteristics	USA	38	0.52
93	1954	Goormaghtigh and Pattyn	A presumably benign tumor and a proved malignant tumor of the carotid body [105]	The American Journal of Pathology	Histopathology/genetics	Belgium	38	0.55
94	1962	Barroso-Moguel and Costero	Argentaffin cells of the carotid body tumor [106]	The American Journal of Pathology	Histopathology/genetics	Mexico	38	0.62
95	1973	Pratt	Familial carotid body tumors [107]	Archives of Otolaryngology	Natural history/disease characteristics	USA	38	0.76
96	1980	Robertson and Cooney	Malignant carotid body paraganglioma: light and electron microscopic study of the tumor and its metastases [108]	Cancer	Histopathology/genetics	Canada	38	0.88
97	2000	Liapis et al.	Role of malignancy and preoperative embolization in the management of carotid body tumors [109]	World Journal of Surgery	Treatment	Greece	38	1.65
98	1982	Larraza-Hernandez et al.	Multiple endocrine neoplasia. Pituitary adenoma, multicentric papillary thyroid carcinoma, bilateral carotid body paraganglioma, parathyroid hyperplasia, gastric leiomyoma, and systemic amyloidosis [110]	American Journal of Clinical Pathology	Histopathology/genetics	Mexico	37	0.90
99	2006	Kollert et al.	Cervical paragangliomas-tumor control and long-term functional results after surgery [111]	Skull Base	Treatment	Germany	37	2.18
100	2013	Sen et al.	Neurological complications in carotid body tumors: a 6-year single-center experience [112]	Journal of Vascular Surgery	Treatment	India	37	3.70

Top 10 Most Cited Articles

The top 10 most cited articles totaled 1741, comprising more than 25% of the total citations. Dates of publication ranged from 1948 to 2007, and they were published in eight different journals. Cancer was the journal with the most publications, observed in three of the top 10. Nine institutions from four countries had the highest impact on the literature on CBTs, with Mayo Clinic, which had two articles, and the United States, Mexico, the United Kingdom, and the Netherlands publishing six, two, one, and one paper(s), respectively. The top 10 articles mainly focused on the natural history/disease characteristics of CBTs, with five articles, followed by treatment and histopathology/genetics, with three and two articles, respectively. The most cited article in this review was an analysis conducted in 1971 by Shamblin and his team from the Mayo Clinic (Table 2).

**Table 2:** The top 10 most cited articles in carotid body tumors #: serial number; YoP: year of publication; CC: citation count; CY: citation per year; INCAN: Instituto Nacional de Cancerologia; USA: United States of America; UK: United Kingdom

#	YoP	Authors	Title	Journal	Institute	Country	Category	CC	CY
1	1971	Shamblin et al.	Carotid body tumor (chemodectoma). Clinicopathologic analysis of ninety cases [4]	The American Journal of Surgery	Mayo Clinic	USA	Histopathology/genetics	462	8.88
2	1950	Lattes	Nonchromaffin paraganglioma of ganglion nodosum, carotid body, and aortic-arch bodies [14]	Cancer	Columbia University	USA	Natural history/disease characteristics	212	2.90
3	1980	Grufferman et al.	Familial carotid body tumors: case report and epidemiologic review [15]	Cancer	Duke University	USA	Natural history/disease characteristics	192	4.47
4	2000	Jansen et al.	Estimation of growth rate in patients with head and neck paragangliomas influences the treatment proposal [16]	Cancer	Leiden University	Netherlands	Natural history/disease characteristics	175	7.61
5	2007	Sajid et al.	A multicenter review of carotid body tumour management [17]	European Journal of Vascular and Endovascular Surgery	Royal Free Hospital	UK	Treatment	153	9.56
6	1995	Netterville et al.	Carotid body tumors: a review of 30 patients with 46 tumors [18]	The Laryngoscope	Vanderbilt University	USA	Treatment	123	4.39
7	1998	Rodríguez-Cuevas et al.	Carotid body tumors in inhabitants of altitudes higher than 2000 meters above sea level [19]	Head & Neck: Journal for the Sciences and Specialties of the Head and Neck	Hospital de Oncología	Mexico	Natural history/disease characteristics	116	4.64
8	2005	Luna-Ortiz et al.	Carotid body tumors: review of a 20-year experience [20]	Oral Oncology	INCAN	Mexico	Natural history/disease characteristics	110	6.11
9	1988	Hallett et al.	Trends in neurovascular complications of surgical management for carotid body and cervical paragangliomas: a fifty-year experience with 153 tumors [21]	Journal of Vascular Surgery	Mayo Clinic	USA	Treatment	101	2.89
10	1948	LeCompte	Tumors of the carotid body [22]	The American Journal of Pathology	Harvard University	USA	Histopathology/genetics	97	1.29

Top 10 Articles in Citation per Year (CY) Metric

The CY metric is calculated by dividing the total CC by the number of years since publication. This is measured to overcome time bias since older articles had more time to collect citations than newer ones. Throughout the 100 included articles, the total CY is 273.55, averaging 2.74 (2.5 standard deviation) per article/per year. The minimum CY is 0.51, while the maximum is 11.71. 

The top 10 articles in this metric were published from 1971 to 2019, with newer articles emerging. The total number of citations is 1275, with an average citation of 127.5 per article, and CY ranged from 6.11 to 11.71, averaging 8.89. The European Journal of Vascular and Endovascular Surgery and Journal of Vascular Surgery each had two of the top 10 most impactful articles in terms of CY, while the rest had one each. Even in CY metric, the Mayo Clinic was the only institution with two publications. Treatment comprised five out of 10 (Table 3).

**Table 3:** The top 10 articles by CY metric #: serial number; YoP: year of publication; CC: citation count; CY: citation per year; INCAN: Instituto Nacional de Cancerologia; USA: United States of America; UK: United Kingdom

#	YoP	Authors	Title	Journal	Institute	Country	Category	CY	CC
1	2016	Moore et al.	Head and neck paragangliomas: an update on evaluation and management [30]	Otolaryngology-Head and Neck Surgery	Indiana University	USA	Review	11.71	82
2	2019	Robertson et al.	A systematic review and meta-analysis of the presentation and surgical management of patients with carotid body tumours [90]	European Journal of Vascular and Endovascular Surgery	The Leicester Vascular Institute	UK	Treatment	11.00	44
3	2017	Williams	Paragangliomas of the head and neck: an overview from diagnosis to genetics [41]	Head and Neck Pathology	University of Texas MD Anderson Cancer Center	USA	Review	10.83	65
4	2007	Sajid et al.	A multicenter review of carotid body tumour management [17]	European Journal of Vascular and Endovascular Surgery	Royal Free Hospital	UK	Treatment	9.56	153
5	1971	Shamblin et al.	Carotid body tumor (chemodectoma). Clinicopathologic analysis of ninety cases [4]	The American Journal of Surgery	Mayo Clinic	USA	Histopathology/genetics	8.88	462
6	2017	Kim et al.	New predictors of complications in carotid body tumor resection [63]	Journal of Vascular Surgery	University of Michigan	USA	Treatment	8.83	53
7	2012	Power et al.	Impact of preoperative embolization on outcomes of carotid body tumor resections [28]	Journal of Vascular Surgery	Mayo Clinic	USA	Treatment	7.64	84
8	2000	Jansen et al.	Estimation of growth rate in patients with head and neck paragangliomas influences the treatment proposal [16]	Cancer	Leiden University	Netherlands	Natural history/disease characteristics	7.61	175
9	2016	Abu-Ghanem et al.	Impact of preoperative embolization on the outcomes of carotid body tumor surgery: a meta-analysis and review of the literature [82]	Head & Neck: Journal for the Sciences and Specialties of the Head and Neck	Tel Aviv University	Israel	Treatment	6.71	47
10	2005	Luna-Ortiz et al.	Carotid body tumors: review of a 20-year experience [20]	Oral Oncology	INCAN	Mexico	Natural history/disease characteristics	6.11	110

Journals

The 100 included articles in this review were published in 41 different journals. The American Journal of Surgery had the highest number of publications, with a total of nine articles (9% of the total articles). It also had the highest CC among the listed journals, with 889 citations with a mean value of 98.78 per article. The highest journal in terms of average citation per article is Cancer, with 100.88. In CY metric, the Journal of Vascular Surgery is by far the highest, with 39.45 CY for their articles. The European Journal of Vascular and Endovascular Surgery holds the most significant average CY per article, with each of their three articles receiving 8.22 citations per year (Table 4).

**Table 4:** Top journals *Journals with two or more articles are listed. #: serial number; CC: citation count; CY: citation per year

#	Journal	Number of articles	CC	Mean	CY	Mean
1	The American Journal of Surgery	9	889	98.78	19.79	2.2
2	Cancer	8	807	100.88	20.1	2.51
3	Journal of Vascular Surgery	8	515	64.38	39.45	4.93
4	Annals of Surgery	8	394	49.25	10.12	1.27
5	The Laryngoscope	6	386	64.3	12.17	2.03
6	British Journal of Surgery	5	327	65.4	8.4	1.68
7	The American Journal of Pathology	5	299	59.8	4.37	0.87
8	Head & Neck: Journal for the Sciences and Specialties of the Head and Neck	3	254	66.23	15.49	5.16
9	European Journal of Vascular and Endovascular Surgery	3	238	79.33	24.66	8.22
10	Otolaryngology-Head and Neck Surgery	3	204	68	20.36	6.79
11	Archives of Surgery	3	170	56.67	4.89	1.63
12	Journal of Neurosurgery	3	137	45.67	3.76	1.25
13	Archives of Otolaryngology	3	131	43.67	2.99	1
14	International Journal of Radiation Oncology*Biology*Physics	2	132	66	5.12	2.56
15	European Archives of Oto-Rhino-Laryngology	2	111	55.5	9.14	4.57
16	World Journal of Surgery	2	82	41	2.91	1.45
17	Surgery	2	95	47.5	1.85	0.92
Total		75				

Authors

The top 100 most cited articles were published by 98 unique first authors. Only two authors, Luna-Ortiz from Mexico and Farr from the United States, appeared as the first authors in two articles each (Table 5).

**Table 5:** First authors with more than one article #: serial number; YoP: year of publication; CC: citation count; CY: citation per year; USA: United States of America

#	YoP	Authors	Title	Journal	Category	CC	C.Y	Country
1	2005	Luna-Ortiz et al.	Carotid body tumors: review of a 20-year experience [20]	Oral Oncology	Natural history/disease characteristics	110	6.11	Mexico
2	2006	Luna-Ortiz et al.	Does Shamblin's classification predict postoperative morbidity in carotid body tumors? A proposal to modify Shamblin's classification [45]	European Archives of Oto-Rhino-Laryngology	Treatment	61	3.59	Mexico
3	1967	Farr	Carotid body tumors. A thirty year experience at Memorial Hospital [55]	The American Journal of Surgery	Treatment	55	0.98	USA
4	1980	Farr	Carotid body tumors: a 40-year study [65]	CA: A Cancer Journal for Clinicians	Review	52	1.21	USA

Categories 

**Figure 1:**
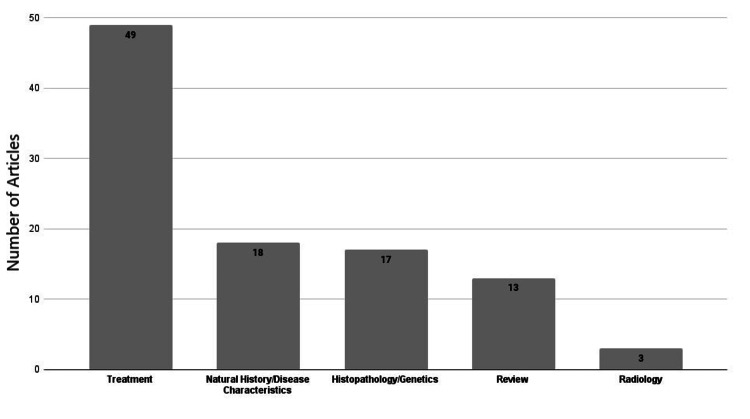
Categories' distribution

The included articles were categorized into five groups, treatment, natural history/disease characteristics, histopathology/genetics, review, and radiology articles, depending on the predominant content and focus of each article. The most notable category extensively discussed in the literature was treatment articles, comprising 49% of all articles (Figure 1).

Institutions

**Figure 2:**
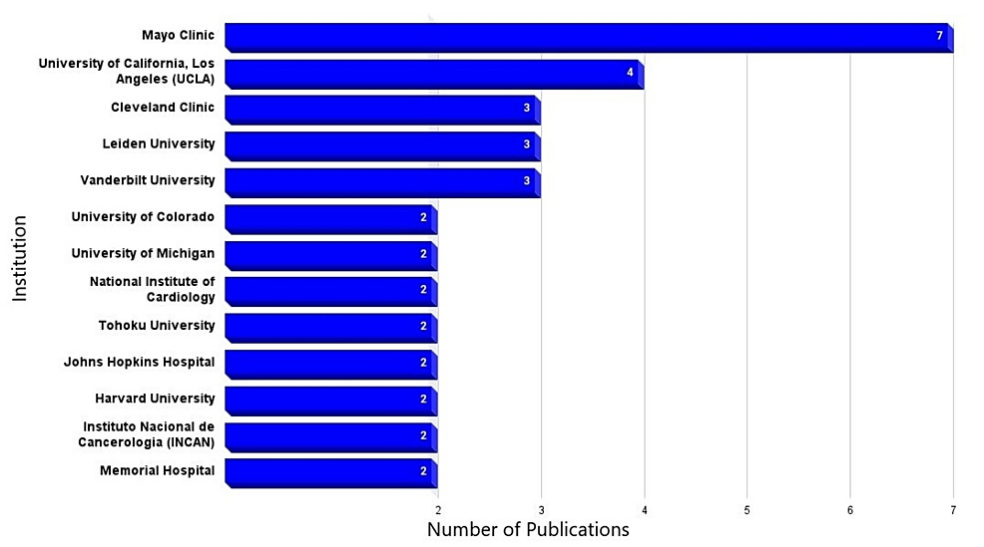
Highest contributing institutions

Seventy-seven institutions contributed to the top 100 most influential articles on CBTs. Of the 77 institutions, 13 had more than one publication in this series. The most significant contributor to the literature was the Mayo Clinic, with seven articles, followed by the University of California, Los Angeles (UCLA), contributing with four articles (Figure 2).

Countries 

**Figure 3:**
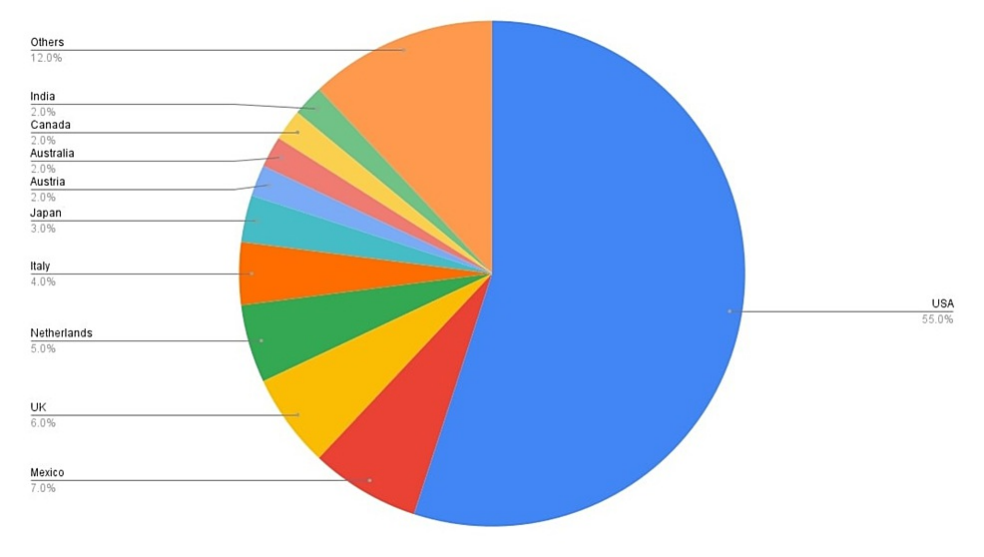
The most prolific countries by contribution

Twenty-two countries were found in this review, and 10 of them had two or more publications. By far the most prolific country is the United States, dominating 55% of the most influential articles on CBTs. Second to the United States, with a significant gap, is Mexico, publishing seven articles. Egypt, Greece, Switzerland, Belgium, France, Ecuador, Spain, Korea, Sweden, Israel, Germany, and South Africa each had one publication only; thus, they were incorporated in "Others" in Figure 3.

Years of Publications

**Figure 4:**
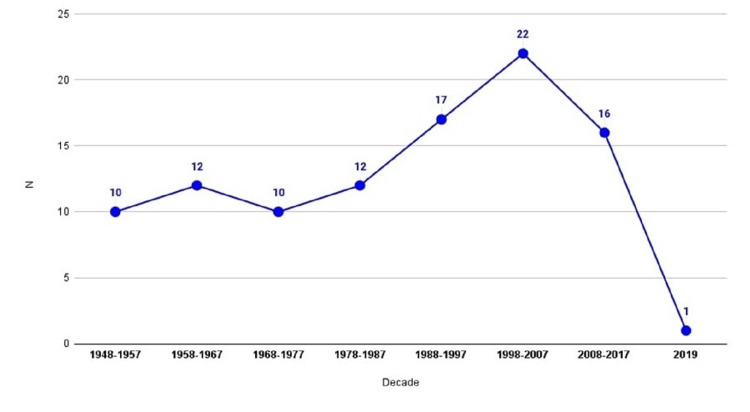
Publication of articles in decades

The top 100 most cited articles were published in a duration of 71 years, from 1948 to 2019. The most prolific decade was 1998-2007, comprising 22 articles (22%) of all included publications, followed by 1988-1997, contributing 17 articles (Figure 4).

Year-to-Year Comparison

**Figure 5:**
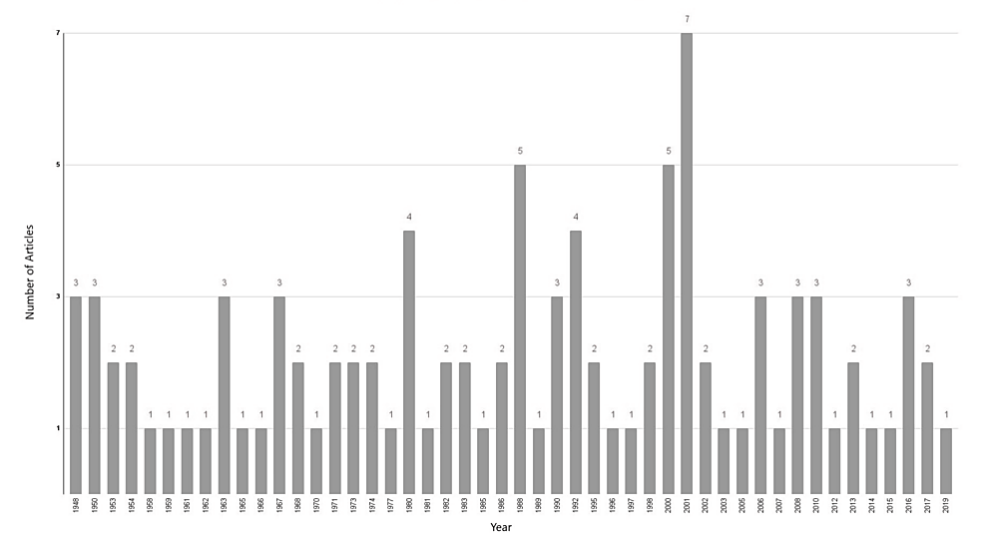
Number of publications per year

The most prolific year was 2001, in which seven articles were published, followed by 2000 and 1988, in which five articles were published (Figure 5).

Discussion

The Most Cited Article

The top-cited article has been cited 462 times as of the time of this writing. It is by far the most influential among the included articles, as there is a 250-citation gap between the first and the second articles. This article is an analysis published in 1971 by a group of four authors led by William R. Shamblin from the Mayo Clinic. In their paper, Shamblin et al. presented a brief review of the carotid body's anatomy, histology, and physiology [4]. The review is followed by a focus on CBTs. At the time, fundamental details of CBTs regarding their behavior and management were unknown. The authors conducted a retrospective analysis from 1931 to 1967 and included 101 CBT patients. Ninety patients fit their inclusion criteria, on whom their research was conducted. The clinical findings, diagnosis, management, follow-up, and histopathology were outlined in detail. Subsequently, they proposed a classification system known as Shamblin groups. Three groups, 1, 2, and 3, were suggested to be used to assess the resectability of tumors and their associated risk. 

Group 1: These are small tumors with minimal attachment to the carotid vessel wall. These tumors are easily dissected from surrounding vessels and can be resected without significant risk of trauma to adjacent structures. 

Group 2: These are larger tumors surrounding the artery and adherent to the arterial wall. The dissection becomes more challenging in these cases. However, careful resection is possible. 

Group 3: These are tumors completely adherent to the carotid bifurcation. In this group, dissection from surrounding structures is not possible. Therefore, approaching these tumors should be under significant care, with vessel replacement considerably needed [4]. 

The Most Cited Article by CY Metric 

The most cited article in the CY metric in this review was conducted by Moore et al., receiving an average of 11.71 every year since 2016. Moore and colleagues, from the Indiana University School of Medicine, published the article "Head and neck paragangliomas: an update on evaluation and management" with the aim of reviewing the updates in diagnostic methods and management strategies, including implementation of radiation therapy and approaching CBTs [30]. This highlights the literature's demand for updated guidelines and reviews as the majority of impactful articles from which practice is derived come from before the 2000s. As demonstrated in Figure 4, the majority (more than 60%) of the articles considered most impactful come from 1948-1997, with the most prolific decade 1998-2007 (Table 3).

Preoperative Embolization

As a subcategory of treatment, approximately 20% of treatment articles (10% of the total articles) discussed preoperative embolization's role in the management of CBTs. Their total CC is 530, comprising 8% of the total citations. Eight out of the 10 papers (80%) were published in the United States, followed by Greece and Israel with one publication each. UCLA was the most active in this subcategory (Table 6).

**Table 6:** Treatment category articles focused on embolization YoP: year of publication; CC: citation count; CY: citation per year; USA: United States of America; UCLA: University of California, Los Angeles

YoP	Author	Article title	Journal name	Institution	Country	CC	CY
1980	Schick et al.	Arterial catheter embolization followed by surgery for large chemodectoma [50]	Surgery	UCLA	USA	57	1.33
1983	Borges et al.	Carotid body tumors managed with preoperative embolization [100]	Journal of Neurosurgery	Harvard University	USA	40	1.00
1988	Ward et al.	Embolization: an adjunctive measure for removal of carotid body tumors [94]	The Laryngoscope	UCLA	USA	41	1.17
1996	Litle et al.	Preoperative embolization of carotid body tumors: when is it appropriate? [23]	Annals of Vascular Surgery	University of California, San Francisco	USA	93	3.44
2000	Liapis et al.	Role of malignancy and preoperative embolization in the management of carotid body tumors [109]	World Journal of Surgery	Athens University	Greece	38	1.65
2001	Kafie and Freischlag	Carotid body tumors: the role of preoperative embolization [84]	Annals of Surgery	UCLA	USA	46	2.09
2010	Zeitler et al.	Preoperative embolization in carotid body tumor surgery: is it required? [91]	Annals of Otology, Rhinology & Laryngology	New York University	USA	43	3.31
2012	Power et al.	Impact of preoperative embolization on outcomes of carotid body tumor resections [28]	Journal of Vascular Surgery	Mayo Clinic	USA	84	7.64
2015	Jackson et al.	The effects of preoperative embolization on carotid body paraganglioma surgery: a systematic review and meta-analysis [97]	Otolaryngology-Head and Neck Surgery	Washington University	USA	41	5.13
2016	Abu-Ghanem et al.	Impact of preoperative embolization on the outcomes of carotid body tumor surgery: a meta-analysis and review of the literature [82]	Head & Neck: Journal for the Sciences and Specialties of the Head and Neck	Tel Aviv University	Israel	47	6.71
Total						530	19.48

The oldest paper included, cited 57 times, which is also the first successful implementation of preoperative embolization was published in 1980 by Schick et al. [50]. The authors gave an insight and introduced this management strategy to the surgical practice by reporting their experience with a 21-year-old man who had a large CBT for five years. They selectively embolized the occipital and posterior auricular arteries and the thyrocervical trunk two weeks prior to the resection. Following embolization, the tumor shrunk by 30%, with 90% of the vascularity occluded. The authors reported that in their experience with this large chemodectoma, surgical dissection was more amenable. Ever since they introduced this practice, many case reports and case series reporting and reviewing this method have been published, catching the interest of the medical community. The conclusion of the said publications mainly suggested that this technique increases the resectability of the tumors and decreases morbidity associated with the surgery [23]. More recently, systematic reviews and meta-analyses on preoperative embolization have been conducted with some conflicting results. One systematic review published by Jackson et al. in 2015 primarily concluded that preoperative embolization decreases operative time and intraoperative blood loss. However, no significant difference was found in the length of hospital stay and complication rates [97]. Another systematic review was published around a year later, in 2016, by Abu-Ghanem et al. had a different conclusion of no significant difference in intraoperative blood loss, operative time, length of hospital stays, or complications [82].

The most influential article in this list, with 93 citations, is a paper titled "Preoperative embolization of carotid body tumors: when is it appropriate?" published in 1996 by Litle and colleagues from the Division of Vascular Surgery at the University of California, San Francisco [23]. In the paper, they published a retrospective study on patients who had CBT resections, spanning 10 years from 1984 to 1994. They categorized tumors into embolized and non-embolized, with 11 in each category. Their review concluded that preoperative embolization of CBTs of midsize, measuring 4-5 cm, does not provide significant improvement in the outcome of patients. Therefore, the authors outlined that as the procedure is costly, it should not be used in midsized tumors. 

Radiology

Three radiology-focused articles were spotted in the most influential articles. They totaled 154 citations (2.3% of the total citations). Italy, the United States, and India each contributed one paper. They all were published in radiology-specialized journals in the years 1992-2008 (Table 7). One focused on the ultrasound evaluation of CBTs, while the remaining focused on the role of MRI in diagnosis and evaluation. Derchi et al. published their paper "Carotid body tumors: US evaluation." They reviewed 20 patients with 23 tumors of the carotid body. They were able to detect CBT in 22/23 tumors in 18/20 patients. They outlined that the US could be utilized as an initial diagnostic modality in suspected CBT cases for its various known advantages, such as noninvasiveness and, as demonstrated in the research, high diagnostic value [81].

**Table 7:** Articles focused on radiological evaluation YoP: year of publication; CC: citation count; CY: citation per year; USA: United States of America

YoP	Authors	Title	Journal name	Institution	Country	CC	CY
1992	Derchi et al.	Carotid body tumors: US evaluation [81]	Radiology	Università di Genova	Italy	47	1.52
2000	Mafee et al.	Glomus faciale, glomus jugulare, glomus tympanicum, glomus vagale, carotid body tumors, and simulating lesions. Role of MR imaging [101]	Radiologic Clinics of North America	University of Illinois	USA	40	1.74
2008	Arya et al.	Carotid body tumors: objective criteria to predict the Shamblin group on MR imaging [37]	American Journal of Neuroradiology	Tata Memorial Hospital	India	67	4.47

For the role of MRI in the evaluation of CBTs, Arya and colleagues from Mumbai, India, published their retrospective review titled "Carotid body tumors: objective criteria to predict the Shamblin group on MR imaging" of nine tumors in eight patients with CBTs spanning the years of 2004-2007. In their paper, they attempted to provide a correlation method between MRI findings and Shamblin groups [37]. 

Impact Analysis 

Categories: The category with the highest impact noted among the five categories is natural history/disease characteristics. The average (mean) CC received by articles in this category is 81.06 (56.75 standard deviation). The least impact was seen by radiology articles. Although there were three articles in this category, the average CC per article was 51.33 (14.01 standard deviation) (Table 8).

**Table 8:** Impact per category CC: citation count

Category		N	Minimum	Maximum	Mean	Std. deviation
Treatment	CC	49	37	153	58.92	23.03
Natural history/disease characteristics	CC	18	38	212	81.06	56.75
Histopathology/genetics	CC	17	37	462	79.53	100.14
Review	CC	13	44	83	59.31	13.34
Radiology	CC	3	40	67	51.33	14.01

Countries and interests: The top 10 countries that contributed to 88% of the literature and dominated the CC have published 88 articles on CBTs. Evidently, the United States dominated the literature both in the number of publications (55%) of influential articles and in the total number of citations, as the vast majority (around 60%) of the citations were of American research articles. Across all high-impact countries, considerable preference towards treatment articles is noted, with 47% of articles treatment-oriented. Next to treatment, the natural history/disease characteristics category comprises 19% of publications (Table 9).

**Table 9:** Cross-tabulation of countries with categories Only countries with more than one publication are included CC: total citation count received by all the papers from the country

			Category					Total
			Histopathology/genetics	Natural history/disease characteristics	Radiology	Review	Treatment	
Country	USA CC: 3934	Count	8	11	1	10	25	55
		% within country	14.55%	20.00%	1.82%	18.18%	45.45%	100.00%
	Mexico CC: 452	Count	3	3	0	0	1	7
		% within country	42.86%	42.86%	0.00%	0.00%	14.29%	100.00%
	UK CC: 496	Count	0	1	0	0	5	6
		% within country	0.00%	16.67%	0.00%	0.00%	83.33%	100.00%
	Netherlands CC: 389	Count	0	1	0	0	4	5
		% within country	0.00%	20.00%	0.00%	0.00%	80.00%	100.00%
	Italy CC: 205	Count	1	0	1	0	2	4
		% within country	25.00%	0.00%	25.00%	0.00%	50.00%	100.00%
	Japan CC: 141	Count	1	1	0	0	1	3
		% within country	33.33%	33.33%	0.00%	0.00%	33.33%	100.00%
	Austria CC: 104	Count	0	0	0	1	1	2
		% within country	0.00%	0.00%	0.00%	50.00%	50.00%	100.00%
	Australia CC: 123	Count	1	0	0	0	1	2
		% within country	50.00%	0.00%	0.00%	0.00%	50.00%	100.00%
	Canada CC: 98	Count	1	0	0	0	1	2
		% within country	50.00%	0.00%	0.00%	0.00%	50.00%	100.00%
	India CC: 104	Count	0	0	1	0	1	2
		% within country	0.00%	0.00%	50.00%	0.00%	50.00%	100.00%
Total CC: 6046		Count	15	17	3	11	42	88
		%	17.05%	19.32%	3.41%	12.50%	47.73%	100.00%

By interest, American research activities were treatment papers, comprising 45.45% of all publications, followed by natural history/disease characteristics. The most influential article is the previously discussed Shamblin paper, "Carotid body tumor (chemodectoma). Clinicopathologic analysis of ninety cases" [4].

The second country in terms of publications is Mexico, with seven articles comprising 452 citations. In contrast to the United States, Mexican articles were equally distributed in the histopathology/genetics and natural history/disease characteristics categories, each comprising 42.86% of articles. The most cited article (116 citations) from Mexico is "Carotid body tumors in inhabitants of altitudes higher than 2000 meters above sea level," published in 1998 by Rodríguez-Cuevas and colleagues. In their research, they investigated the differences between CBTs occurring in patients living in high altitudes, defined as 2200 meters above sea level, and patients living in low altitudes. The study did find that cases of CBTs in a high-altitude population have differences from those in populations living at lower altitudes. High-altitude cases have a higher prevalence in females, a lower rate of bilateral tumors, and a lower incidence of family history compared to those living in low-altitude areas in the United States or Europe. The female-to-male ratio is 8.3:1 in high-altitude cases versus 2:1 in low-altitude cases. Bilaterality is found in 5% of high-altitude cases compared to 10-20% in low-altitude cases. Finally, a family history of CBT is present in only 1% of high-altitude cases compared to 7-25% in low-altitude cases [19]. 

Third in rank comes the United Kingdom, with six articles and 496 citations. Similar to the United States, the categorical interest is patently the treatment articles, comprising 83.33% of papers, with one on the natural history/disease characteristics category. The most influential article, with 153 citations, is titled "A multicenter review of carotid body tumour management," published by Sajid and his colleagues from Royal Free Hospital in 2007. The aim of their study was to provide significant evidence by conducting the largest series on CBTs in the European region at the time. They outlined, by analyzing 95 patients from 10 centers, the diagnostic challenges, surgical intervention, and complications of CBTs. Their analysis found that although surgical intervention is indicated in most cases, it is not without risks. The associated morbidity of surgical resection reaches 33-35%, mainly cranial nerve injury, bleeding, transient ischemic attacks (TIA)/strokes, and Horner syndrome, and a mortality of 1% [17].

The only Arab countries noted in this bibliometric analysis are Egypt and Saudi Arabia. Thabet and Kotob, who were affiliated with Alexandria University (Egypt) and Fakhry and Al Mouhawis Hospital (Saudi Arabia), published the article "Cervical paragangliomas: diagnosis, management and complications" in 2001 in The Journal of Laryngology & Otology. In their paper, they retrospectively studied 16 patients, 11 of whom had CBTs, from the above institutions from 1990 to 1999. They outlined details of the included cases, such as locations, family tendencies, secretory activities, size of the tumors, and diagnostic methods. Operative findings of the included paraganglioma with anatomical descriptions are outlined. Complications of surgical resection in the form of neurological deficits were reported in 27% (3/11) of patients. Vascular injuries were reported in 9% of patients (9/11) [95]. 

Limitations

This bibliometric review has some limitations that should be considered. First, articles with high CC do not necessarily signify a significant impact on this specific topic, as some studies may have been cited to demonstrate a weakness or for actions of criticism. Also, CC is subject to bias since older studies had more time than recent ones to receive citations. In attempting to alleviate this limitation, the CY metric was used in our review. 

In addition, this review used one database to search for articles: WoS. This means other impactful studies may have been missed if not indexed in the WoS. Also, the CC is calculated as per WoS journals. This means that citations from journals outside of WoS may have been missed. However, WoS covers the oldest publications as opposed to other search engines such as Google Scholar, Scopus, and PubMed. The data on WoS goes back to 1900. Its content consists of 8700 journals, as compared to 6000 covered by PubMed. Also, the citation analysis of WoS provides more details than Scopus [113].

## Conclusions

This analysis of the top 100 most cited articles on CBTs offers key insights into the current research landscape and highlights the importance of ongoing high-quality research to advance the understanding, diagnosis, and treatment of this condition. While identifying influential articles, authors, institutions, countries, and journals, the study also highlights a predominant focus on treatment and natural history/disease characteristics, with limited attention to radiology. Moving forward, there is a need for future research to not only address these gaps but also explore the integration of radiological imaging modalities for improved diagnosis and treatment planning. Additionally, emphasis should be placed on collaborative efforts among researchers and clinicians to further develop innovative therapeutic strategies that can enhance patient outcomes. These recommendations aim to guide and inspire future studies in this field, ultimately contributing to advancements in patient care and management.
